# Development of a Real-Time PCR Assay to Identify and Distinguish between *Cryptococcus neoformans* and *Cryptococcus gattii* Species Complexes

**DOI:** 10.3390/jof8050462

**Published:** 2022-04-29

**Authors:** Enoch Tay, Sharon C-A. Chen, Wendy Green, Ronald Lopez, Catriona L. Halliday

**Affiliations:** 1 Research Education Network, Western Sydney Local Health District, Westmead Hospital, Westmead, NSW 2145, Australia; enoch.tay@health.nsw.gov.au; 2 Centre for Infectious Diseases and Microbiology Laboratory Services, Institute of Clinical Pathology and Medical Research, NSW Health Pathology, Westmead Hospital, Westmead, NSW 2145, Australia; sharon.chen@health.nsw.gov.au (S.C.-A.C.); wendy.green@health.nsw.gov.au (W.G.); ronald.lopez@health.nsw.gov.au (R.L.)

**Keywords:** *Cryptococcus neoformans*, *Cryptococcus gattii*, *Cryptococcus* PCR, diagnosis of cryptococcosis

## Abstract

*Cryptococcus neoformans* and *Cryptococcus gattii* are the principle causative agents of cryptococcosis. Differences in epidemiological and clinical features, and also treatment, mean it is important for diagnostic laboratories to distinguish between the two species. Molecular methods are potentially more rapid than culture and cryptococcal antigen (CRAG) detection; however, commercial PCR-based assays that target *Cryptococcus* do not distinguish between species. Here, we developed a real-time PCR assay targeting the multicopy mitochondrial cytochrome *b* (*cyt b*) gene to detect *C. neoformans* and *C. gattii* in clinical specimens. Assay performance was compared with culture, histopathology, CRAG and panfungal PCR/DNA sequencing. The *cyt b*-directed assay accurately detected and identified all eight *C. neoformans*/*gattii* genotypes. High-resolution melt curve analysis unambiguously discriminated between the two species. Overall, assay sensitivity (96.4%) compared favorably with panfungal PCR (76.9%) and culture (14.5%); assay specificity was 100%. Of 25 fresh frozen paraffin embedded (FFPE) specimens, assay sensitivity was 96% (76% for panfungal PCR; 68% for histopathology). The *Cryptococcus*-specific PCR is a rapid (~4 h) sensitive method to diagnose (or exclude) cryptococcosis and differentiate between the two major species. It is suitable for use on diverse clinical specimens and may be the preferred molecular method for FFPE specimens where clinical suspicion of cryptococcosis is high.

## 1. Introduction

Cryptococcosis, caused by the *Cryptococcus neoformans*-*Cryptococcus gattii* complex, is a life-threatening fungal disease of global importance with significant attributable mortality (20.2% and 28.4% for *C. gattii* and *C. neoformans*, respectively) [1]. *C. neoformans* is found worldwide and typically causes opportunistic infection in patients with underlying immunodeficiency, while *C. gattii* is considered more geographically restricted and typically, although not exclusively, affects immunocompetent hosts [1]. Both species are species complexes and can each be further divided into two serotypes and four molecular types (*C. neoformans*, serotypes A and D and VNI-IV, and *C. gattii*, serotypes B and C and VGI-IV) [2]. Differences in ecology, epidemiology, clinical manifestations and treatment approaches emphasize the importance of distinguishing between the two species complexes [3,4].

Laboratory diagnoses of cryptococcosis are based on a combination of histopathology, direct microscopy, fungal culture and/or a positive cryptococcal antigen (CRAG) test [4,5]. While culture is generally sensitive and simple to perform, culture-negative infections can occur. The detection of CRAG in serum has high sensitivity and specificity for the diagnosis of central nervous system (CNS) cryptococcosis, but may be less sensitive for the diagnosis of pulmonary (only) infection [6]. Additionally, neither CRAG detection nor microscopy allow the speciation of *Cryptococcus* and CRAG measurement is only applicable to serum and cerebral spinal fluid (CSF). Molecular methods offer a potentially more rapid diagnostic approach to identify *C. neoformans* and *C. gattii* and to differentiate between their molecular types, but methods are not standardized and evaluations have primarily been applied to DNA extractions from pure cultures only [7,8].

A number of commercial, syndromic-based PCR-based panels for CNS and respiratory infections (Biofire meningitis/encephalitis (ME) panel (bioMérieux, Marcy l’Etoile, France) and Multiplex Tandem PCR (MT-PCR) Atypical Pneumonia and CSF panels (AusDiagnostics, Mascot, Australia)) include *Cryptococcus*-specific targets for diagnostic use [9,10,11]. These assays target the internal transcribed spacer (ITS) region, but are only applicable to certain specimen types and importantly, do not differentiate between *C. neoformans* and *C. gattii* [10,12,13]; further, several studies have reported false negatives on culture-proven cases [14,15,16]. Alternate gene targets for potential incorporation into PCR assays include the fungal mitochondrial (mt) cytochrome *b* gene (*cyt b*), known to be useful for the identification and classification of yeasts such as *Candida* spp. [17,18]. Biswas et al., in a proof-of-concept study, showed sufficient discrimination by this locus for identifying all the major molecular types of *C. neoformans* species complex [19]. However, there are few data on the use of *cyt b*-targeted PCR assays in detecting *Cryptococcus* species for diagnostic purposes.

Our laboratory presently relies on panfungal PCR approaches followed by DNA sequencing to detect and identify *Cryptococcus* DNA in specimens such as bronchoalveolar lavage fluid (BALF), fresh and formalin-fixed, paraffin-embedded (FFPE) tissue, which is technically challenging, particularly when taken from non-sterile specimens [20,21], and slow. To facilitate the timely diagnosis of the main agents of cryptococcosis, here we developed a targeted real-time PCR assay to detect and identify *C. neoformans* and *C. gattii* directly from clinical specimens. We chose to target the *cyt b* gene, as it is multicopy (to maximize sensitivity) with sufficient homology to detect both the *C. neoformans* and *C. gattii* complexes using a single primer pair, yet has sufficient variation to differentiate between the species complexes by melt curve analysis [19]. The assay was first tested on isolates of the major molecular types of *C. neoformans* complex and then using a range of archived clinical specimens in a laboratory-based evaluation.

## 2. Materials and Methods

### 2.1. Control DNA and Clinical Specimens

DNA extracts representing the eight major molecular types of *C. neoformans* (VNI, ATCC MYA-4564/WM148; VNII, ATCC MYA-4565/WM626; VNIII, ATCC MYA-4566/WM628; and VNIV, ATCC MYA-4567/WM629) and *C. gattii* (VGI, ATCCMYA 4560/WM179; VG II, ATCC MYA-4561/WM178; VGIII, ATCC MYA-4562/WM161; and VGIV, ATCC MYA-4563/WM779) were provided by Prof. Wieland Meyer, Molecular Mycology Research Laboratory (Centre for Infectious Diseases and Microbiology, Westmead Institute for Medical Research, Westmead, Australia). To evaluate the specificity of the assay, DNA from the following fungal pathogens was tested: *Aspergillus calidoustus*, *Aspergillus flavus*, *Aspergillus fumigatus*, *Aspergillus terreus*, *Candida albicans*, *Cryptococcus laurentii*, *Cryptococcus magnus*, *Fusarium solani*, *Lomentospora prolificans*, *Mucor circinelloides*, *Paecilomyces variotti*, *Penicillium* spp., *Purpureocillium lilacinum*, *Rhizopus microsporus*, *Scedosporium apiospermum*, *Talaromyces* spp. and *Trichosporon asahii*. These species were selected based on their local prevalence in the clinical specimens being tested and/or their genomic similarity. All the strains included in the study were identified by sequencing the ITS region of the rDNA. Archived clinical specimens (*n* = 208), collected between January 2018 and June 2021 submitted for routine culture as well as panfungal PCR from patients with documented cryptococcosis (*n* = 55) or alternate pathogens (*n* = 153), were tested; all were collected as part of the routine standard of care for examination of fungi and were stored in our laboratory. Specimens from patients with confirmed cryptococcosis included FFPE tissue (*n* = 25), CSF (*n* = 14), BALF (*n* = 6), fine needle aspirates (FNA) (*n* = 5), induced sputum (IS) (*n* = 3) and unspecified (*n* = 2).

### 2.2. DNA Extraction

Specimen manipulations and DNA extractions were performed in a class II biological safety cabinet. DNA from cultures and clinical specimens was extracted using either the NucliSens^®^ EasyMag^®^ (bioMérieux) or the High Pure PCR Template Preparation kit (Roche Diagnostics Australia Pty Ltd., North Ryde, Australia) according to the manufacturer’s instructions for the High Pure PCR Template Preparation kit, including the addition of MagNA Lyser Green Beads (Roche Diagnostics Australia Pty Ltd.), bead beating of tissue samples and a final elution volume of 80 µL for all specimen types [20]. DNA was stored at −20 °C prior to use.

### 2.3. Primer and Probe Design

Sequences of the *cyt b* gene of the *C. neoformans* (IFM5505, IFM5854, IFM58844, IFM46090) and *C. gattii* (IFM5815, IFM48634, IFM5856 and IFM5875) complexes were aligned using Clustal Omega [22] to determine regions of homology and diversity for primer and probe design. The primers amplify an 82-base pair (bp) product. The probe sequence has complete homology to *C. neoformans* and differs at two nucleotides to *C. gattii* sequences. The two species can be differentiated by post-PCR high-resolution melt (HRM) analysis, where variations in the *C. neoformans* and *C. gattii* sequences result in melting temperatures of 81 °C and 79 °C, respectively. Primer and probe sequences are shown in Table 1. Additionally, synthetic, double-stranded gene fragment sequences (“gBlocks^®^”, Integrated DNA Technologies, Coralville, IA, USA) were designed as positive control DNA and contained the *cyt b* gene from either *C. neoformans* or *C. gattii* (Table 1).

**Table 1:** Primer, probe and positive control sequences for the *Cryptococcus* real-time PCR assay.

Species Primer and Probe	Sequence (5′-3′)
***Cryptococcus neoformans-gattii*** **complex**	
*Cryptococcus cyt b* F	TTCTAGCAGCTCTAGCTCTAG
*Cryptococcus cyt b* R	GCATTTGAGCTAATACCTTCAGG
*Cryptococcus cyt b* probe	6FAM-TACATATGCTAACACTTCACACACA-BHQ1
**Human β globin** **(internal control)** [23]	
HBG F	GAAGAGCCAAGGACAGGTAC
HBG R	CACCAACTTCATCCACGTTCAC
HBG probe	TxRd-TCAAACAGACACCATGGTGCACCTG-BHQ2
**Positive Control DNA**	
*C. neoformans* (82 bp)	TTCACTATCTACTACCATTTATTCTAGCAGCTCTA-GCTCTAGTACAATGCTAACACTTCACACACACGGTAGCTCAAACCCTGAAGGTATTAGCTCAAATGC-TGAAAAGGCACCAATGCATCCATACTTTA
*C. gattii* (82 bp)	TTCACTATCTACTACCATTTATTCTAGCAGCTCTA-GCTCTAGTACATATGCTAACACTACACTCACAT-GGTAGTTCAAATCCTGAAGGTATTAGCTCAAAT-GCAGAAAAGGCACCAATGCATCCATACTTTA

*cyt b*, mitochondrial cytochrome *b*; HBG, human β globin.

### 2.4. Multiplex Real-Time PCR and High-Resolution Melt (HRM) Curve Analysis

PCRs were performed in a 25 µL final volume containing 2× HotStarTaq^®^ master mix (Qiagen, Hilden, Germany), 0.5 µM *Cryptococcus cyt b* forward and reverse primers, 0.1 µM *Cryptococcus cyt b* probe, 0.2 µM of the Human β globin (HBG) forward and reverse primers and probe [23], and 10 µL DNA extract or water for the no template control. The PCR amplification was conducted using a LightCycler 480 II instrument (Roche Diagnostics, Mannheim, Germany) as follows: 10 min of pre-denaturation at 95 °C, followed by 45 cycles of 95 °C for 10 s, 55 °C for 30 s and 72 °C for 15 sec, with single acquisitions made after each 55 °C stage, and a final cooling cycle of 40 °C for 30 s. Each experiment was performed in duplicate, with the replicate excluding HBG primers and probe to be used for HRM analysis if the sample yielded a positive *Cryptococcus* result. Results were considered positive when they yielded a cycle threshold (CT) ≤45. On completion of the PCR, positive samples were subjected to HRM analysis by adding 25 µL of 2× SensiFAST^TM^ SYBR^®^ No-ROX mix (Meridian Bioscience, Cincinnati, OH, USA) to each positive sample well (without HBG amplification). The PCR products were subjected to 0.14 °C/s ramping between 72 °C and 87 °C with four acquisitions per second. Synthetic double-stranded *C. neoformans* and *C. gattii* DNA targets (Table 1) were included as positive control standards for the melting peaks, to determine if the melting peak of a clinical sample represents *C. neoformans* or *C. gattii*.

The PCR assay was first validated using the DNA of the eight molecular genotypes of *C. neoformans*/*C. gattii* before the testing of archived clinical specimens. For all specimens, assay performance was compared with results from fungal culture, CRAG, histology and panfungal (ITS-directed) PCR and DNA sequencing, where available.

## 3. Results

### 3.1. PCR Specificity and Sensitivity

**Figure 1:**
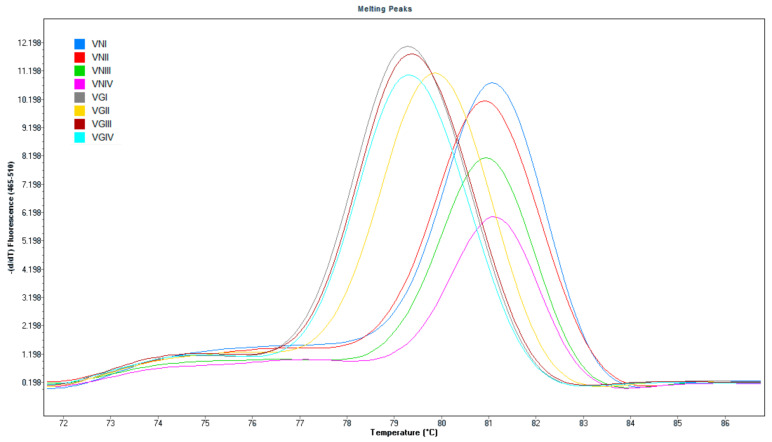
Melt curves of amplification products from four molecular subtypes of *Cryptococcus neoformans* (right) and four molecular subtypes of *Cryptococcus gattii* (left) at 81 °C and 79 °C, respectively. The molecular types are depicted using different colors in the figure.

The *cyt b*-directed assay detected all eight molecular genotypes of *C. neoformans* and *C. gattii* with a limit of detection of ~10^1^ colony-forming units (CFU)/mL. HRM analysis demonstrated *C. neoformans* (VNI-VNIV) and *C. gattii* (VGI-VGIV) isolates had a melting point of 81 °C and 79 °C, respectively (Figure 1). The assay cannot discriminate between individual molecular types within each species as the melting points are too close. No amplification was obtained from any of the other fungal or bacterial species tested.

### 3.2. PCR Performance on Clinical Specimens

Using the in-house assay developed here, 53/208 (25.4%) specimens tested positive for either *C. neoformans* (*n* = 38) or *C. gattii* (*n* = 15) (Table 2). PCR-negative specimens (*n* = 155) encompassed a range of specimens, including IS and BALF submitted for *Pneumocystis jirovecii* infection and CSF specimens referred for testing on the MT-PCR CSF panel (AusDiagnostics).

The assay had an overall sensitivity of 96.4% (53/55) with only two specimens (FFPE and CSF) from patients with cryptococcosis testing PCR-negative. Table 2 summarizes the performance of the *Cryptococcus cyt b* assay with other diagnostic modalities. The PCR enabled diagnosis and species identification in 24/25 (sensitivity 96%) FFPE tissue samples compared with histology (17/25; sensitivity 68%) and culture (0/25); panfungal PCR and DNA sequencing achieved a diagnosis in 19/25 (sensitivity 76%) cases. Additionally, there were two specimens (BALF and FFPE) where both the *Cryptococcus*-targeted and panfungal PCR assays detected the presence of cryptococcal DNA; however, the panfungal PCR was only able to assign an identification of *C. neoformans-gattii* complex, while the targeted assay and HRM analysis could assign a species identification to both.

For fresh clinical specimens (BALF, IS, and CSF FNA), the average turn-around-time (TAT) from DNA extraction to issuing a negative or positive *Cryptococcus* PCR report, including species identification, was 4 h; FFPE specimens require additional processing prior to DNA extraction and the TAT for these specimens was ~48 h. The TAT for the panfungal PCR and DNA sequencing was between 72 and 108 h for fresh and FFPE specimens.

## 4. Discussion

The identification of *C. neoformans* and *C. gattii* to the species level is important to guide treatment regimens and inform clinical progression and outcomes [1,3,4]. Although the differentiation of the two species from culture-positive clinical specimens can be performed reliably by matrix-assisted laser desorption ionization-time of flight mass spectrometry, inoculation onto canavanine-glycine-bromothymol (CGB) agar [24,25], or a variety of molecular methods, including multi-locus sequence typing, PCR fingerprinting and amplified fragment length polymorphism analysis [7,8], these approaches are either slow (up to several days) and/or require access to laboratories performing genotyping methods. In the present study, we present the results using an in-house sensitive *Cryptococcus*-specific real-time PCR assay, to not only allow the ready identification of the causative species from diverse sterile and non-sterile site specimen types, but to provide a more rapid TAT and cost savings. This has the potential to replace the panfungal PCR assay currently performed in the laboratory, with beneficial workflow implications.

We first validated the specificity of the *Cryptococcus*-specific assay using authenticated reference strains. Although the assay was not intended *per* se to discriminate between all cryptococcal genotypes, it could *detect* all eight molecular genotypes and HRM analysis was able to discriminate between *C. neoformans* and *C. gattii* without ambiguity (Figure 1). A global study of the molecular epidemiology of *Cryptococcus* found that in Australia the prevalent genotypes are VNI (131/164) and VNII (25/164) for *C. neoformans* and VGI (179/300) and VGII (121/300) for *C. gattii* [26]. Whilst the relevance of detecting known genotypes remains uncertain in clinical practice [4], global travel and immigration from regions where other genotypes are more frequent make it important to have an all-encompassing approach for a diagnostic assay. Of note, we designed the assay to directly detect and distinguish between *C. neoformans* and *C. gattii* in clinical specimens, because this addresses the major clinical need.

When tested on a diverse range of stored clinical specimens, the *Cryptococcus*-PCR assay and HRM analysis was 100% specific and 96.3% sensitive for detecting and identifying *C. neoformans* and *C. gattii*. For the FFPE specimens, the molecular diagnosis was consistent with the histological diagnosis where available (19/25 cases). The two specimens that tested negative on the targeted assay were a CSF and FFPE. The successful amplification of fungal DNA from FFPE tissue is dependent on a number of factors, including the amount of fungi in the tissue, the amount of tissue available for DNA extraction and the length of time the tissue has been fixed in formalin, which affects the integrity of the DNA and leads to smaller fragments [27]. It is plausible that these two specimens which tested negative contained fungal DNA amounts that were at the limit of detection for the assay. Of note, histopathology demonstrated no fungal elements on the FFPE sample, despite the finding of a positive serum CRAG with a titer of 640. In the case of the CSF specimen, encapsulated yeast cells were seen on India ink stain and, although the CSF CRAG was positive, the titer was only 10, suggesting a low fungal load.

Overall, the *Cryptococcus*-specific assay was more sensitive than the panfungal assay, which had a sensitivity of 76.9% (40/52). In our laboratory, IS specimens are not a suitable specimen type for the panfungal assay. Our panfungal PCR assay targets both the ITS1 and ITS2 regions in independent reactions to increase the likelihood of assigning a species identification to positive samples [20]. However, despite the ITS region being able to discriminate between *C. neoformans* and *C. gattii* [28], two of the 40 positive samples could not be confidently distinguished and were identified as *C. neoformans–gattii* complex. This may be due to sequence quality and/or the ITS regions being amplified independently, rather than in a single assay inclusive of the 5.8*S* rRNA gene. Samples that were negative on the panfungal assay and positive on the *Cryptococcus*-targeted assay included FFPE tissue (*n* = 6), CSF (*n* = 3) and BALF (*n* = 3). Two of six FFPE specimens had encapsulated yeast cells seen in histology; the results were unavailable for the remaining four. Panfungal PCR testing on BALF specimens is particularly problematic due to the presence of multiple fungal species and/or commensal fungi with reported diagnostic yields as low as 4.5–23% when performed in unselected patients [21,29,30]. The targeted approach has good potential to overcome this limitation and, for practical purposes, we found it to be accurate for both sterile and non-sterile site specimens.

The *Cryptococcus*-specific assay was able to diagnose (or exclude) cryptococcosis in patients within four hours, improving the TAT by >3 days compared with positive panfungal PCR and/or culture. The assay was also able to offer cost savings of USD 40 per sample compared with the panfungal PCR assay and ≥USD 180 compared with the BioFire ME panel, which, at USD 220/test [31], would preclude its routine use in most Australian laboratories. Unlike both the panfungal and commercial PCR assays (BioFire ME and AusDiagnostics Atypical Pneumonia and CSF) and CRAG, the targeted assay is applicable to a diverse range of clinical specimens, including respiratory tract specimens which often contain other fungal flora, and FFPE tissue which may not have been referred for microbiological culture. We envisage that with more clinical experience, the assay will obviate the need to perform panfungal PCR and DNA sequencing for provision of a diagnosis of cryptococcosis from these specimens, where the index of suspicion cryptococcosis is high. This would offer considerable financial savings as well as a shorter time to diagnosis.

## Figures and Tables

**Table 2:** Results of *Cryptococcus*-specific PCR compared with other diagnostic modalities.

Specimen Type (*n*)	Culture Positive	CRAG	Histology	Panfungal PCR	Targeted PCR *C. neoformans*	Targeted PCR *C. gattii*
FFPE tissue (25)	N/A	N/A	13	19	20	4
CSF (14)	2	3	N/A	11	7	6
BALF (6)	3	N/A	N/A	3	2	4
FNA (5)	2	N/A	N/A	5	4	1
IS (3)	0	N/A	N/A	N/A	3	0
Not Specified (2)	1	N/A	N/A	2	2	0
**TOTAL (55)**	**8**	**3**	**13**	**40**	**38**	**15**

BALF, bronchoalveolar lavage fluid; CRAG, cryptococcal antigen; CSF, cerebral spinal fluid; FFPE, formalin fixed paraffin-embedded; FNA, fine needle aspirate; N/A, not applicable; IS, induced sputum.

## Data Availability

Sequence data available on request.
